# Cancer-associated fibroblast-derived exosomal miR-18b promotes breast cancer invasion and metastasis by regulating TCEAL7

**DOI:** 10.1038/s41419-021-04409-w

**Published:** 2021-12-01

**Authors:** Ziqian Yan, Zhimei Sheng, Yuanhang Zheng, Ruijun Feng, Qinpei Xiao, Lihong Shi, Hongli Li, Chonggao Yin, Hao Luo, Chong Hao, Wenhao Wang, Baogang Zhang

**Affiliations:** 1 Department of Pathology, Weifang Medical University, Weifang, Shandong China; 2 Department of Pathology, Affiliated Hospital of Weifang Medical University, Weifang, Shandong China; 3 Department of Pharmacology, Weifang Medical University, Weifang, Shandong China; 4 Department of Medicine Research Center, Weifang Medical University, Weifang, Shandong China; 5 Department of Oncology, Maternal and Child Health Care Hospital of Zibo, Zibo, Shandong China; 6 Department of Medical Oncology, Affiliated Hospital of Weifang Medical University, Weifang, Shandong China

**Keywords:** Breast cancer, Single-molecule biophysics

## Abstract

Studies have shown that cancer-associated fibroblasts (CAFs) play an irreplaceable role in the occurrence and development of tumors. Therefore, exploring the action and mechanism of CAFs on tumor cells is particularly important. In this study, we compared the effects of CAFs-derived exosomes and normal fibroblasts (NFs)-derived exosomes on breast cancer cells migration and invasion. The results showed that exosomes from both CAFs and NFs could enter into breast cancer cells and CAFs-derived exosomes had a more enhancing effect on breast cancer cells migration and invasion than NFs-derived exosomes. Furthermore, microRNA (miR)-18b was upregulated in CAFs-derived exosomes, and CAFs-derived exosomes miR-18b can promote breast cancer cell migration and metastasis by specifically binding to the 3′UTR of Transcription Elongation Factor A Like 7 (TCEAL7). The miR-18b-TCEAL7 pathway promotes nuclear Snail ectopic activation by activating nuclear factor-kappa B (NF-κB), thereby inducing epithelial-mesenchymal transition (EMT) and promoting cell invasion and metastasis. Moreover, CAFs-derived exosomes miR-18b could promote mouse xenograft model tumor metastasis. Overall, our findings suggest that CAFs-derived exosomes miR-18b promote nuclear Snail ectopic by targeting TCEAL7 to activate the NF-κB pathway, thereby inducing EMT, invasion, and metastasis of breast cancer. Targeting CAFs-derived exosome miR-18b may be a potential treatment option to overcome breast cancer progression.

## Introduction

Breast cancer is one of the common malignant tumors among women worldwide. Recurrence and metastasis are the main causes of death in breast cancer patients [1], and the tumor microenvironment has a more significant impact on the development and metastasis of tumors.

CAFs are active fusiform or polygonal fibroblasts with highly heterogeneous and contractile characteristics [2]. CAF may affect cancer cells and other stromal cells by releasing high levels of factors such as growth factors, cytokines, chemokines, and metalloproteinases [3–5]. Thus, CAFs can promote tumor progression and metastasis by providing mechanical support or by using paracrine, direct interaction, and enhancing the chemical resistance of tumor cells [6].

Exosomes are vesicle corpuscles with lipid bilayer structures secreted by various somatic cells and tumor cells [7]. With a diameter of about 40−100 nm, exosomes contain miRNAs, RNAs, proteins, and other small molecular substances and have attracted extensive attention as an important medium of intercellular communication [8–10]. MiRNAs are single-stranded non-coding RNAs of about 20 nucleotides in length. They can specifically bind to post-transcriptional mRNA to regulate gene expression leading to degradation of target genes or inhibition of protein expression [11]. miRNAs can influence the occurrence and development of tumors by regulating oncogenes or anti-oncogenes [12–15]. miR-18b associate with the drug resistance and tumor formation of multiple tumors [16, 17]. However, the detailed role of CAF-derived exosomes carrying miR-18b in breast cancer remains unclear.

As a transcriptional regulator on X chromosome, TCEAL7 was first discovered by Chien et al. through transcriptional cloning [18]. TCEAL7 is a member of transcription elongation factor A (SII) -like genes family that contain TFA domains and can act as nuclear phosphoproteins that regulate transcription in a promoter context-dependent manner [19]. The TCEAL7 gene encodes a cell death regulator protein containing 100 amino acids and is homologous in sequence with TCEAL1, TCEAL6, and brain-expressed (Bex) [20]. TCEAL7 is downregulated in multiple tumors and functions as a tumor suppressor [21], such as non-small cell lung cancer [22]. However, the detailed role of TCEAL7 has not been thoroughly clarified in breast cancer.

In this study, we found that TCEAL7 is negatively regulated by miR-18b, in vivo, and in vitro experiments have demonstrated that exosomal miR-18b can promote the migration and invasion of breast cancer cells by targeting the expression of TCEAL7.

## Materials and methods

### Patient samples and ethical statement

The tumor tissues of the breast cancer patients came from the affiliated hospital of Weifang Medical University. During surgery, ten pairs of matched tumor tissues and adjacent normal tissues were collected to isolate CAFs and NFs, and washed fresh cancer and normal tissues with PBS containing 20% antibiotics immediately. Isolation of CAFs and NFs by immunomagnetic bead cell sorting (MACS) [23]. After washing the specimen, cut it into small pieces, add DMEM/F-12 medium containing 0.5% collagenase IV and 1% FBS and incubate the cell suspension for 30 min. The primary medium for fibroblasts (Dulbecco’s modified DMEM/F-12 medium containing 10% fetal bovine serum). The primary fibroblasts cultured from two to three generations were digested, counted, and resuspended in MACS buffer. Anti-human fibroblast magnetic beads were added and purified in an immunomagnetic bead cell sorter. The purified CAFs and NFs were cultured in DMEM medium containing 10% FBS, and cells from 4 to 10 passages were used for experiments. The moral approval comes from the system ethics committee of Weifang Medical University. The written informed consent of all subjects participating in the study was obtained.

### Cell culture

Breast cancer cell lines MCF-7, MDA-MB-231 were obtained from the American Type Culture Collection (ATCC, Manassas, VA), and cultured in RPMI-1640 medium containing 10% fetal bovine serum (FBS). The cell line has recently been authenticated by STR profiling, and also were tested for mycoplasma contamination every six months and identified as negative. Primary fibroblasts and cancer-associated fibroblasts were grown in Dulbecco modified DMEM/F-12 medium containing 10% FBS. The cell lines were kept at 37 °C in 5% CO_2_ humidification atmosphere and logarithmic growth phase cells were used for the experiment. To prepare exosome depleted FBS, the supernatant was extracted under aseptic conditions by 110,000 × *g* supernatant centrifugation for 18 h at 4 °C. In experiments involving exosomes, exosome depleted FBS was used in cell culture.

### Isolation of exosomes

After 24 h of culture in serum-free medium, the supernatant of normal fibroblasts and cancer-associated fibroblasts culture was collected. The cells were incubated with the complete medium containing serum to a density of about 80%, discarded the supernatant, and continued culture with serum-free medium. More than 10 mL of cell culture supernatant was collected after 48 h. The residual cells and debris were removed by centrifugation at 3,000 × *g* for 15 min at 4 °C. Transfer the supernatant to a new centrifuge tube and add 2 mL of ExoQuick-TC™ Exosome Isolation Reagent (SBI, System Bioscience) for every 10 mL of culture medium. Then agitate the tube slightly until the sample is entirely mixed. After storing at 4 °C for 12 h, centrifuge at 5,000 × *g* for 30 min, discard the supernatant, and the precipitate is exosomes.

### Western blotting

Protein was isolated by SDS-PAGE gel and transferred to PVDF membrane. After 1 h of blocking in 5% skim milk, the membrane was incubated with various primary antibodies and incubated overnight at 4 °C with Anti-Alix (sc-53540, Santa Cruz), Anti-α-SMA (ab7817, Abcam), Anti-CD63 (ab134045, Abcam), Anti-FAP (ab207178, Abcam), Anti-ICAM1 (ab282575, Abcam), Anti-MMP-9 (ab76003, Abcam), Anti-MMP-3 (ab52915, Abcam), Anti-β-catenin (ab32572, Abcam), Anti-Ki67 (ab15580, Abcam), Anti-PCNA (ab92552, Abcam), Anti-MMP-2 (ab92536, Abcam), Anti-PDGFR beta (ab69506, Abcam), Anti‐β‐actin (A5441, Sigma), Anti-E-cadherin (SAB4503751, Sigma), Anti-N-cadherin (C3865, Sigma), Anti-Vimentin (V6389, Sigma), Anti-snail (SAB1306281, Sigma), Anti-Zeb1 (SAB2500097, Sigma), Anti-Zeb2 (SAB3500515, Sigma), Anti-Slug (PRS3959, Sigma), Anti‐HSP70 (4873, Cell Signaling Technology), Anti‐GM130 (G7295, Sigma), Anti-FUS (DF8391, Affinity), Anti-ACO1 (AF4711, Affinity), Anti-YTHDC1 (77422, Cell Signaling Technology), Anti-TCEAL7 (DF9969, Affinity). And after three times of elution with TBST, the secondary antibody linked with horseradish peroxidase was incubated for 1 h and the electrochemical luminescence was observed by chemiluminescence apparatus. Results quantization was performed by ImageJ software.

### Transmission electron microscopy (TEM)

The separated exosomes were fixed with 4% paraformaldehyde and then dripped into the formaldehyde-coated electron microscope grid and fixed with 1% glutaraldehyde for 10 min. The sample was stained with 1% uranyl-oxalate solution for 5 min. Removing excess liquid and images were obtained using JEOL 1011 transmission electron microscope at 60 kV.

### Nanoparticle tracking analysis

Nanosight LM10 system (Nanosight Ltd, Navato, CA) was used to analyze the size and density of exosomes. The system was equipped with fast video capture and particle tracking software. The Brownian motion of the nanoparticles is captured by video, and the process is repeated three times, and the Brownian motion rate was measured by NTA analytical software to calculate the concentration and size distribution of the nanoparticles.

### Cell transfection

A lentiviral plasmid encoding miR-18b/mimics or inhibitor, TCEAL7, and its siRNA and negative control was designed and produced by Genechem (Shanghai, China). According to the instructions, Lipofectamine 2000 reagent (Invitrogen, California, USA) was used to transfect breast cancer cells. GW4869 (Sigma, California, USA) was used to inhibit exosomal release.

### RNA extraction and reverse transcriptase quantitative real-time PCR (qRT-PCR)

All operations were performed according to the manufacturer’s instructions. TRIzol reagent (Invitrogen; Thermo Fisher Scientific, Inc) isolated total RNA from cells and exosomes, and the RevertAid First Strand cDNA synthesis kit (Thermo Fisher Scientific, Inc) was used for cDNA synthesis. The expression of mature miRNAs was detected by real-time quantitative PCR (qPCR) using SYBR Premix (Perfect Real Time) kit. All of the reactions were run in triplicate. U6 snRNA was used as an internal control of miRNAs, and mRNA levels were normalized against β-actin. The cycle threshold (C_T_) data were determined by fixed threshold settings, and the average *C*_T_ was determined from triplicate PCRs. The data analysis employed 2^−ΔΔCt^ method.

### Bioinformatics analysis

Meta-analysis of global gene expression data in the Oncomine database (Compendia Bioscience, Ann Arbor, USA) was performed using primary filters for “breast cancer” and “cancer vs normal analysis”, data type filter to use “mRNA” data sets and sample filter to use “clinical specimens” (10 datasets representing 2941 patients). Patients include patients of all ages, genders, disease stage, or treatment. Data were acquired in an unbiased manner by compiling all the Oncomine studies with significantly altered TCEAL7 expression at the threshold settings (*P*-value = 0.05, foldchange = 2, and gene rank = all). Significant studies in which at least one analyzed group was comprised of three patients or less were excluded. In the Oncomine database, all data are reported as log2 median-centered intensity. Export the dataset from Oncomine and analyze it in GraphPad Prism V7 software.

### Migration and invasion

In vitro invasion experiments were performed by 6.5 mm Transwell 24-well plates. Add 600 μL of RPMI-1640 medium containing 10% FBS in the lower chamber, and coat the upper chamber with 25 μL of Matrigel Basement Membrane Matrix (BD) and serum-free RPMI-1640 mixed solution (configure scale bit 1: 6), and dry at 37 °C at least 4 h. Then implant 120,000 cells in the upper chamber and incubate for 16 h in the incubator. Wash three times with PBS, fix with 4% paraformaldehyde, and stain Giemsa for 15 min. Rinse off the staining solution with slow running water and leave to dry. Scanned under a microscope after sealing with resin.

### DiD labeling of exosomes

Vybrant DiD (Life Technologies) was used to label exosomes according to the manufacturer’s instructions [24]. In brief, DiD and PBS were diluted at 1:1000 and incubated at room temperature for 10 min. Exosomes were then re-purified using a qEV Size Exclusion Columns (Izon Science Ltd.).

### Immunofluorescence analysis

The cells were fixed with 4% paraformaldehyde, 0.5% TritonX-100 was infiltrated for 15 min, and washed three times with PBS. Blocking with BSA for 1 h, and incubate at 4 °C with the primary antibody for 1 h in the dark, then incubated with Alexa Fluor 568 (Invitrogen, California, USA) at 37 °C for 1 h in the dark with the secondary antibody, stained with DAPI (Sigma, California, USA). Finally, the cells were observed with a confocal fluorescence microscope.

### Luciferase assays

The 3′UTR region (5′-CCAUCUGUAUAAAAACACCUUG-3′) of TCEAL7 targeted by miR-18b was screened by TargetScan software. 3′UTR terminal mutation reporting vector was designed and constructed by Shanghai GenePharma Co.,Ltd. According to the instructions of Lipofectamine 2000, the luciferase reporter vector was used to co-transfected MDA-MB-231 cells with miR-18b, miR-18b inhibitor, miR-18b mutant, and control sequence, and then the cells were put into an incubator at 37 °C and 5% CO_2_ for 48 h. Luciferase activity was tested by a detector.

### Immunohistochemistry (IHC)

The tissue was embedded in paraffin fixed with formalin, dewaxed with xylene and dehydrated with ethanol, and then extracted with 0.01 mm citrate buffer (pH 6.0). The Rabbit anti-α-SMA monoclonal antibody was incubated overnight at 4 °C, and anti-rabbit secondary antibody was incubated at 37 °C for 30 min. Finally, it was developed with DAB and counterstained with hematoxylin. Taking photos under an optical microscope.

### Immuno-precipitation (IP)

Immuno-precipitation using anti-FUS antibody was performed at 48 h after treatment. Briefly, cells were collected and lysed by the lysis buffer. Then Lysates were centrifuged at 14,000 × *g* for 15 min. The supernatant was mixed with FUS antibody (Affinity) overnight at 4 °C, and then co-cultured with beads (Santa Cruz) for incubated at 4 °C for 4 h. The beads were washed five times in lysis buffer and then subjected to Western blot analysis.

### In vivo tumor growth and metastasis experiment

The prepared BALB/c nude female mice (4–5 weeks, *n* = 10; Charles River) were randomly divided into two groups (*n* = 5). One group of MDA-MB-231 cells were floating in 150 mL of PBS and injected into the mice through the tail vein, and the other group was injected with cancer cells co-cultured with exosomes miR-18b. Thirty days later, the tumor growth was monitored using biofluorescence imaging technology, and then the mice were sacrificed, and the lung tissue was removed for observation and photographing. Our analysis did not exclude any data. All experimental procedures involving mice were performed following the Guidelines for the Care and Use of Experimental Animals and were approved by the Animal Care and Use Research Committee of Weifang Medica University.

### Statistical analysis

All data were analyzed by SPSS 17.0 statistical software. Each experiment was repeated for three times, and the comparison of measurement data was conducted by independent sample t-test and paired sample t-test. All data were meet normal distribution, and the variance among the groups statistically compared was similar. The difference among groups was analyzed by one-way analysis of variance (ANOVA). Mean ± SD was used for quantitative data, and *p* < 0.05 was considered statistically significant.

## Results

### CAFs promotes the migration and invasion of breast cancer cells

**Fig. 1:**
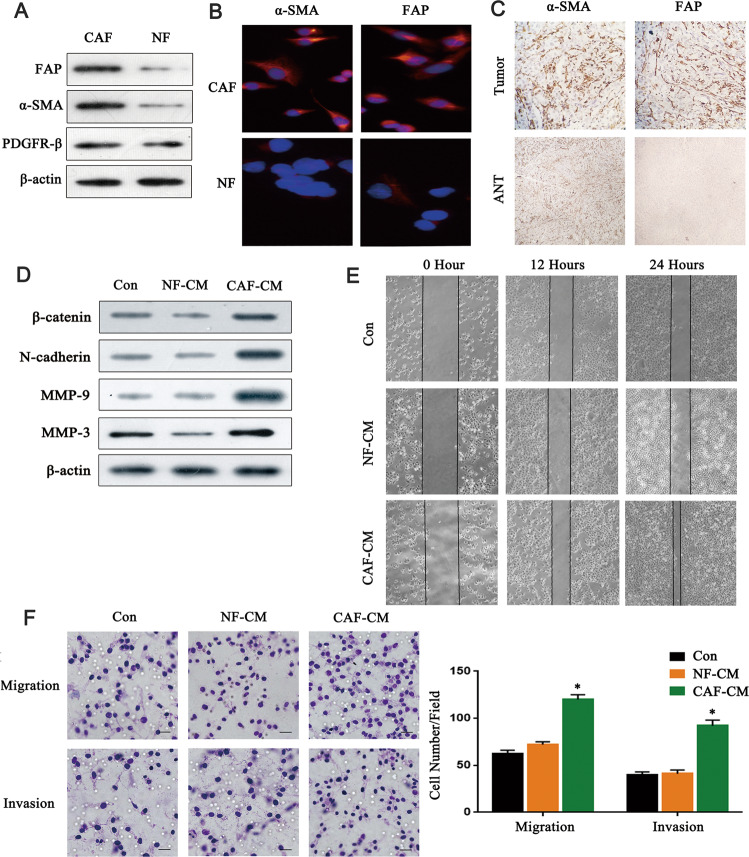
CAFs promote the migration and invasion of breast cancer cells. **A** Western blot analysis of FAP, α-SMA, and PDGFR-β proteins in isolated fibroblasts (*n* = 3). **B** Immunofluorescence analysis detected the expression of α-SMA and FAP in isolated fibroblasts. Scale bar, 50 μm. **C** Immunohistochemical staining analysis of α-SMA and FAP expression in breast cancer tissues and adjacent normal tissues. Scale bar, 200 μm. **D** MDA-MB-231 breast cells were co‑cultured with NF-CM or CAF-CM. The protein of MDA-MB-231 breast cells was extracted and detected the expression of β‑catenin, N-cadherin, MMP‑9, and MMP‑3 by western blot. **E** The effect of CAFs and NFs on the migration of breast cancer cells was analyzed by scratch experiment. **F** Transwell invasion assays analyzed the effection of CAFs and NFs on the invasion ability of breast cancer cells (*n* = 3). Scale bar, 30 μm. * indicates *p* < 0.05. The measurement data were expressed using mean ± SD, and the experiment was repeated three times; one-way ANOVA was used for multiple groups of data analysis.

We first isolated CAFs and NFs from BC (breast cancer) tissues and adjacent normal tissues. Western blot results showed that the expression levels of α-SMA and FAP in CAFs were higher than those in NFs. Still, there was no significant difference in the expression of PDGFR-β between the two groups (Fig. 1A). Immunofluorescence staining and immunohistochemical staining were used to further verify the higher expression of α-SMA and FAP in CAFs or breast cancer tissues (Fig. 1B, C). To evaluate the effect of isolated CAFs on cancer cells, we treated MDA-MB-231 breast cancer cells with NF-CM and CAF-CM, and then detected several metastasis-related proteins. As shown in Fig. 1D, there is no significant difference between NF-CM groups and control groups whereas MDA-MB-231 breast cells co-cultured with CAF-CM showed increased expression of β‑catenin, N-cadherin, MMP-9, and MMP-3, indicating that CAF-CM elevated the migration and invasion capacity of the breast cancer cells. The scratch test and Transwell showed no significant difference between NF-CM groups and control groups whereas MDA-MB-231 breast cancer cells co-cultured with CAF-CM showed higher migration and invasion capacity (Fig. 1E, F), and there are similar results in MCF-7 cells (Supplementary Fig. S1A, B). (*P* < 0.05). These findings indicated that CAFs could promote the migration and invasion of breast cancer cells.

### CAFs derived exosomes enhance migration and invasion of breast cancer cells

**Fig. 2:**
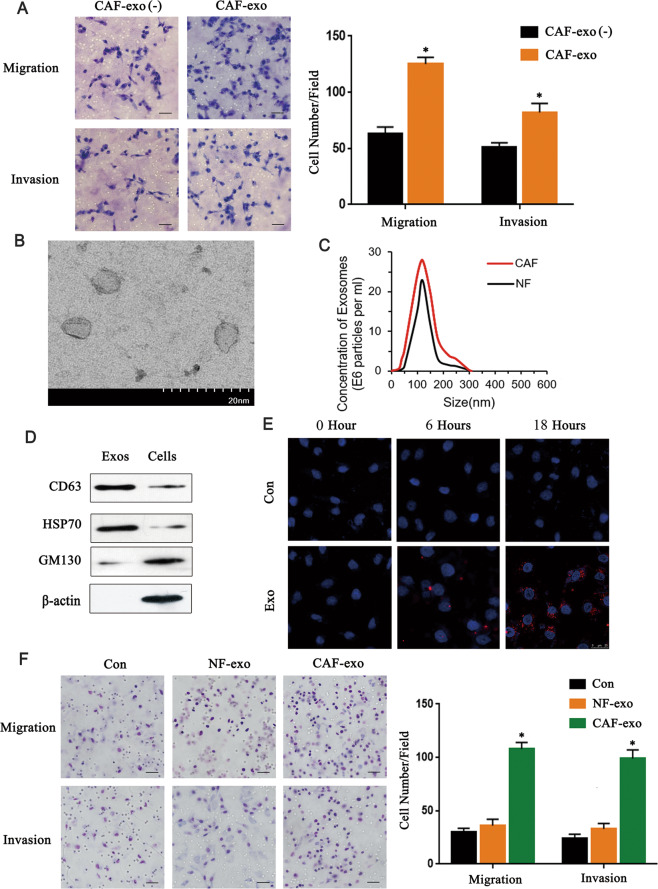
CAFs derived exosomes enhance invasion and migration of breast cancer cells. **A** Transwell experiments analyzed the effects of exosomes on the invasion and migration ability of breast cancer cells (*n* = 3). Scale bar, 50 μm. **B** Electron microscopic image of exosomes isolated. Scale bar, 20 nm. **C** The particle size distribution and counts were evaluated by Tunable Resistive Pulse Sensing (*n* = 3). **D** Western blot detected the expression of exosomes and cells markers in CAFs-derived exosomes and fibroblasts (*n* = 3). **E** Immunofluorescence imaging shows DiD-labeled exosomes and cell uptake of exosomes (*n* = 3). Scale bar, 25 μm. **F** The effects of exosomes from different fibroblast sources on the invasion and migration ability of breast cancer cells were analyzed by Transwell assays (*n* = 3). Scale bar, 50 μm. * indicates *p* < 0.05. The measurement data were expressed using mean ± SD, and the experiment was repeated three times. Comparisons between two groups are analyzed by paired t-test; one-way ANOVA was used for multiple data analysis groups.

Exosomes play an important role in intercellular communication. We considered whether exosomes from CAFs promote the migration and invasion of breast cancer cells. We cultured MDA-MB-231 cells in CAF-CM with exosome depletion and CAF-CM containing exosomes respectively and found that the conditioned medium containing exosomes significantly promote the migration and invasion of cells (*P* < 0.05) (Fig. 2A), and there are similar results in MCF-7 cells (Supplementary Fig. S2A). Then exosomes were isolated from the CM of NFs and CAFs and electron microscopy showed that the isolated exosomes displaying typical lipid bilayer morphology ranging in size from 30 to 150 nm (Fig. 2B, C). In addition, the exosome expressed exosome protein markers CD63 and Hsp70 (Fig. 2D) which confirmed that we got purified exosomes from both CAF-CM and NF-CM. CM-DiD labeled CAFs-derived exosomes were co-cultured with MDA-MB-231 cells, and the distribution of CM-DiD in cells was analyzed by immunofluorescence which showed that exosomes could be uptaken into cancer cells (Fig. 2E). And Transwell showed that CAFs-derived exosomes could promote migration and invasion of MDA-MB-231 cells more obviously than that of NFs-derived exosomes (*P* < 0.05) (Fig. 2F), and there are similar results in MCF-7 cells (Supplementary Fig. S2B). Collectively, these data indicated that exosomes released by CAFs promote the migration and invasion of breast cancer cells.

### The upregulated expression of miR-18b in exosomes enhanced migration and invasion of breast cancer cells

**Fig. 3:**
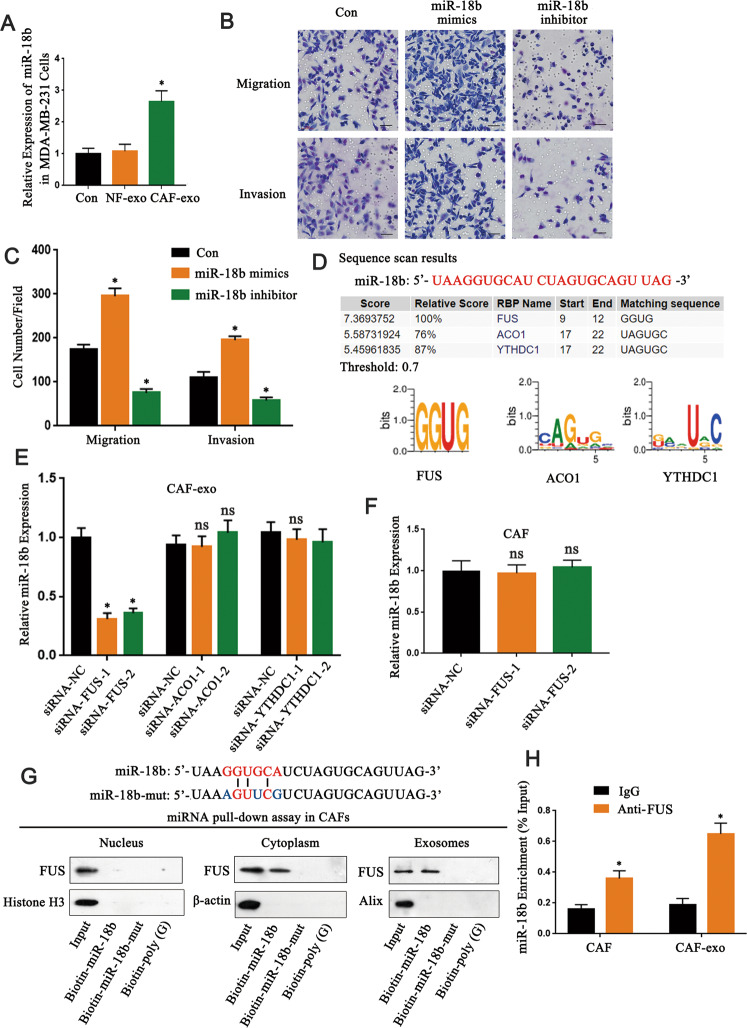
The upregulated expression of miR-18b in exosomes enhanced migration and invasion of breast cancer cells. **A** qRT-PCR detected miR-18b expression in cancer cells co-cultured with NFs and CAFs-derived exosomes (*n* = 3). **B**, **C** Transwell experiments demonstrated the effect of miR-18b on the ability of breast cancer cells in migration and invasion (*n* = 3). Scale bar, 50 μm. **D** RBPDB analysis was used to predict the specific interaction between the miR-18b sequence and RBP motifs (threshold 0.7). **E** Using real-time PCR to measure the expression of miR-18b in CAFs-derived exosomes transfected with specific siRNA targeting FUS, ACO1, or YTHDC1 (*n* = 3). **F** Real-time PCR analysis detected the expression of miR-18b after silencing FUS in CAFs (*n* = 3). **G** The miRNA pull-down experiment analyzed the interaction of miR-18b and miR-18b mutant with FUS in the cytoplasm, nucleus, and exosomal lysates of CAFs, biotinylated poly. **H** As a negative control (*n* = 3). **I** Anti-FUS antibody (or IgG as a control) was used for RIP detection of CAFs cells or exosomal lysates (*n* = 3). * indicates *p* < 0.05. The measurement data were expressed using mean ± SD, and the experiment was repeated three times. Comparisons between two groups are analyzed by t-test; one-way ANOVA was used for multiple data analysis groups.

The transfer of exosomes containing miRNAs between cells is considered to be a new important mechanism of genetic exchange [25]. miRNA-expression datasets related to breast cancer GSE134022 were retrieved from GEO database (https://www.ncbi.nlm.nih.gov/geo/) for differential analysis of miRNA expression (Supplementary Fig. S3A). 39 upregulated miRNAs and 20 downregulated miRNAs were identified with log fold change (|logFC | ) > 1.5 and *p* value < 0.05 as the screening threshold. In order to identify the highest miRNA in CAF-derived exosomes, we evaluated the 10 upregulated miRNAs in CAF- and NF-derived exosomes by real-time PCR. The results showed that miR-18b-5p (also known as miR-18b) was the most upregulated miRNA in the CAF-derived exosomes (*P* < 0.05) (Supplementary Fig. S3B). miR-18b has been reported to be abnormally expressed in many tumors [16, 17, 26], therefore, it could be an important target for cancer treatment. The expression of miR-18b in breast cancer cells co-cultured with CAFs-derived exosomes was higher than that of NF-derived exosomes (*P* < 0.05) (Fig. 3A; Supplementary Fig. S4A). We further found that the miR-18b mimics significantly enhanced migration and invasion capacity of MDA-MB-231 cells (*P* < 0.05) whereas the miR-18b inhibitors have the contrary effects (*P* < 0.05) (Fig. 3B, C), and there are similar results in MCF-7 cells (Supplementary Fig. S4B). In addition, we detected the cell proliferation rate in both miR-18b mimics/MDA-MB-231 and Con/MDA-MB-231 cells in vitro. The same number of Con/MDA-MB-231 cells and miR-18b mimics/MDA-MB-231 cells were simultaneously plated, and cell numbers were counted from the next day. The proliferation assay result shows that increased miR-18b did not result in significant changes in cell proliferation (*P* < 0.05) (Supplementary Fig. S5). These data emphasized the importance of miR-18b in CAFs derived exosomes in the migration and invasion of breast cancer cells.

### The packaging of miR-18b into exosomes is mediated by FUS

In order to explore the mechanism of miR-18b being specifically packaged into exosomes, we analyzed the specific interaction between miR-18b sequence and RNA binding protein (RBPs) motifs through the RBP specific database (RBPDB, http://rbpdb.ccbr.utoronto.ca/; threshold 0.7). The results showed that fused in sarcoma (FUS), aconitase 1 (ACO1), and YTH domain containing 1 (YTHDC1) motifs have specific miR-18b binding sites (Fig. 3D). Further research revealed that knockdown of FUS in CAFs by specific siRNAs significantly reduced exosome miR-18b levels (*P* < 0.05) while intracellular miR-18b levels remained almost unchanged (*P* > 0.05) (Fig. 3E, F). This indicated that FUS plays a regulatory role of miR-18b in exosomes. The miRNA pull-down analysis suggested that the interaction between FUS and miR-18b can be observed in the cytoplasm and exosomes, but cannot be detected in the nucleus. However, FUS binding ability was impaired when the GGUGCA sequence of miR-18b was mutated (Fig. 3G). In addition, subsequent RNA immunoprecipitation (RIP) analysis of CAFs cells and exosome lysates revealed that miR-18b was enriched in the FUS antibody group compared to the IgG group (*P* < 0.05) (Fig. 3H). The above data demonstrated that FUS might play an important role in packaging miR-18b into exosomes by binding a specific motif (GGUG) of miR-18b, thus FUS-mediated miR-18b enrichment in exosomes might play an active role in breast cancer progression.

### miR-18b reduce the expression of TCEAL7 by binding to its 3′UTR

**Fig. 4:**
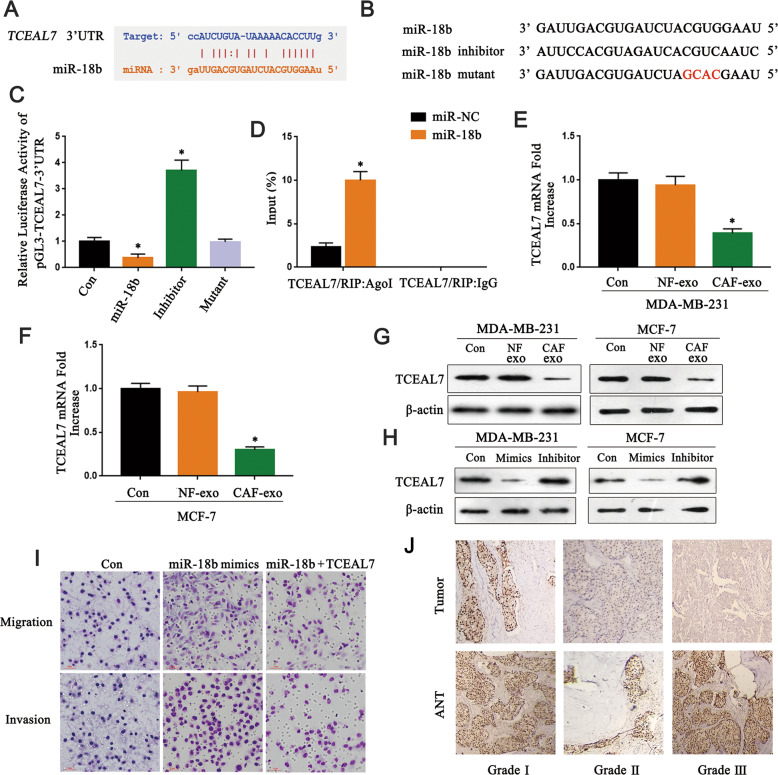
miR-18b binds to the 3′UTR region of TCEAL7 to reduce its expression. **A** Targetscan predicted that miR-18b combined with TCEAL7. **B**, **C** The target relationship between miR-18b and TCEAL7 verified by luciferase reporter gene assays. **D** Immunoprecipitation experiment to verify the binding of TCEAL7 and miR-18b (*n* = 3). **E**, **F** qRT-PCR showed that the expression of TCEAL7 was downregulated after co-cultured with CAFs-derived exosomes and NFs-derived exosomes (*n* = 3). **G** Western blot verified the results of qRT-PCR (*n* = 3). **H** Western blot observed the expression of TCEAL7 after transfected with miR-18b mimics and inhibitor in cancer cells (*n* = 3). **I** MDA-MB-231 cells are transfected with NC mimics+NC, miR-18b mimics+NC, or miR-18b mimics+pcDNA-TCEAL7 to detect invasion and migratory cell number by transwell. **J** Immunohistochemistry showed TCEAL7 expression in breast cancer tissues with different pathological grades (*n* = 3). * indicates *p* < 0.05. The measurement data were expressed using mean ± SD, and the experiment was repeated three times. Comparisons between two groups are analyzed by nonpaired t-test; one-way ANOVA was used for multiple data analysis groups.

**Fig. 5:**
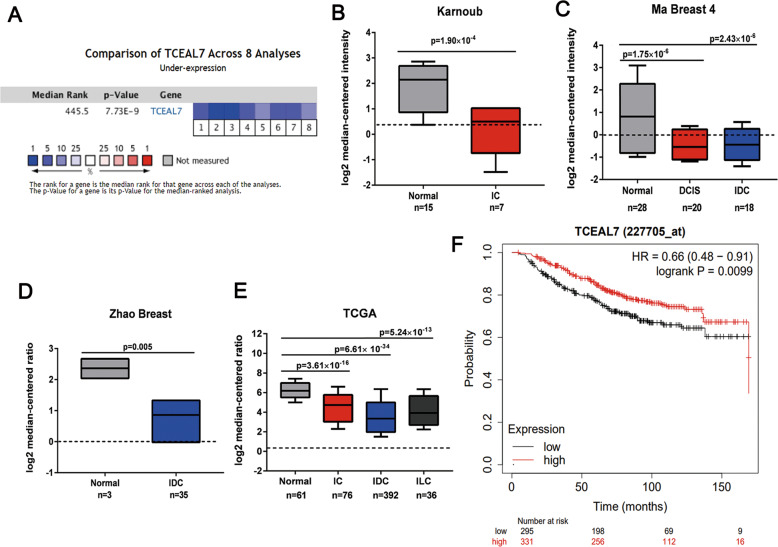
TCEAL7 expression is reduced in human breast cancers. **A** Meta-analysis data of TCEAL7 differential expression in breast cancers vs. normal breast tissues. Oncomine microarray database was used to analyze TCEAL7 mRNA expression and meta-analysis was performed on eight analyses from 10 microarray datasets (2941 patients). Data are shown as median rank of TCEAL7 expression through each dataset analysis. *P*-value for TCEAL7 was determined by using the median ranked analysis of breast cancer vs. normal tissues. 1. Invasive Ductal Breast Carcinoma Stroma vs. Normal, Karnoub Breast, Nature, 2007; 2. Ductal Breast Carcinoma in Situ Stroma vs. Normal, Ma Breast 4, Breast Cancer Res, 2009; 3. Invasive Ductal Breast Carcinoma Stroma vs. Normal, Ma Breast 4, Breast Cancer Res, 2009; 4. Ductal Breast Carcinoma vs. Normal, Richardson Breast 2, Cancer Cell, 2006; 5. Invasive Breast Carcinoma vs. Normal, TCGA Breast, No Associated Paper, 2011; 6. Invasive Ductal Breast Carcinoma vs. Normal, TCGA Breast, No Associated Paper, 2011; 7. Invasive Lobular Breast Carcinoma vs. Normal, TCGA Breast, No Associated Paper, 2011; 8. Invasive Ductal Breast Carcinoma vs. Normal, Zhao Breast, Mol Biol Cell, 2004. **B**−**E** Differential expressions of TCEAL7 mRNA in the 10 datasets included in the meta-analysis (Normal: normal adjacent breast tissue; IC: invasive breast carcinoma; IDC: invasive ductal breast carcinoma; DCIS: Ductal carcinoma in situ; ILC: invasive lobular breast carcinoma). Median and interquartile range (10th and 90th percentiles). Two-sided t-test for two class differential expression analyses and Pearson’s correlation for multiclass analyses. FDR-corrected *P*-values. **F** Kaplan−Meier plots showing the overall survival for TCEAL7 expression (probe: 227705_at). Log-rank *P*-values calculated in kmplot database.

To further disclose the mechanism of exosomes miR-18b promoting tumor cells invasion and metastasis, the 3’UTR region (5′-CCAUCUGUAUAAAAACACCUUG-3′) of TCEAL7 targeted by miR-18b was screened by TargetScan software (Fig. 4A). In addition, luciferase assay demonstrated that miR-18b was complementary to the 3′UTR region of TCEAL7 (*P* < 0.05) (Fig. 4B, C). RIP analysis showed that mRNAs of TCEAL7 from MDA-MB-231 cells transfected with miR-18b could be specifically adsorbed by miRNP complex isolated by anti-AgoI antibody (*P* < 0.05) (Fig. 4D). PCR and Western blot results suggested that CAF-derived exosomes suppressed the MDA-MB-231 cells and MCF-7 cells expression of TCEAL7 (*P* < 0.05) (Fig. 4E, G). Interestingly, the more reduced TCEAL7 expression was observed in the miR-18b mimics group, and the reduced TCEAL7 expression was rescued in the miR-18b inhibitor group (Fig. 4H). Rescue experiments illustrated that TCEAL7 was able to abolish the regulatory effects of miR-18b on the invasion and migratory abilities of breast cancer cells (Fig. 4I). The decreased expression level of TCEAL7 was negatively correlated with pathological grade (Fig. 4J) (Sample clinical information is shown in Table 1). To further verify the low expression of TCEAL7 in human breast cancers, a meta-analysis of publicly available gene expression data was performed using the Oncomine database. We compared TCEAL7 expression in BC with normal adjacent BC samples from ten datasets and found underexpressed TCEAL7 in BC samples (gene median rank: 445.5, *P* = 7.73e−9) in eight datasets included in the meta-analysis (Fig. 5A). Conformably, reduced TCEAL7 mRNA levels were also found in BC samples including invasive breast carcinoma, invasive ductal breast carcinoma, Ductal carcinoma in situ, invasive lobular breast carcinoma as compared with the corresponding normal breast tissues (*P* < 0.05) (Fig. 5B, E). In addition, Kaplan-Meier analysis on breast cancer patients stratified by TCEAL7 mRNA levels showed that low TCEAL7 mRNA level (probe 227705_at) is correlated with lower overall (Fig. 5F) survivals than patients with high TCEAL7 mRNA levels. Therefore, these in silico data suggest the reduced TCEAL7, level in human BC which is related to a worse prognosis. Collectively, we proved that miR-18b efficaciously inhibited the expression of TCEAL7 in human BC through binding to its 3′UTR region.

**Table 1:** Association between TCEAL7 expression and clinical features of breast cancer patients.

Variables	TCEAL7 expression		*p* value
	High expression	Low expression	
Age (years)			
≤50	75	42	0.130
≥51	78	65	
Tumor size (cm)			
≤5 cm	71	50	0.705
å 5 cm	85	54	
Tumor differentiation			
I	57	32	
II	64	41	0.046
III	52	14	
Lymph node metastasis			
Yes	98	53	0.022
No	55	54	
Distant metastasis			
Yes	94	49	0.031
No	61	56	
Estrogen receptor			
Positive	82	68	0.374
Negative	67	43	
Progesterone receptor			
Positive	76	64	0.619
Negative	69	51	
CerbB-2			
Positive	80	45	0.101
Negative	72	63

### TCEAL7 reverse EMT by suppressing the NF-κB pathway

**Fig. 6:**
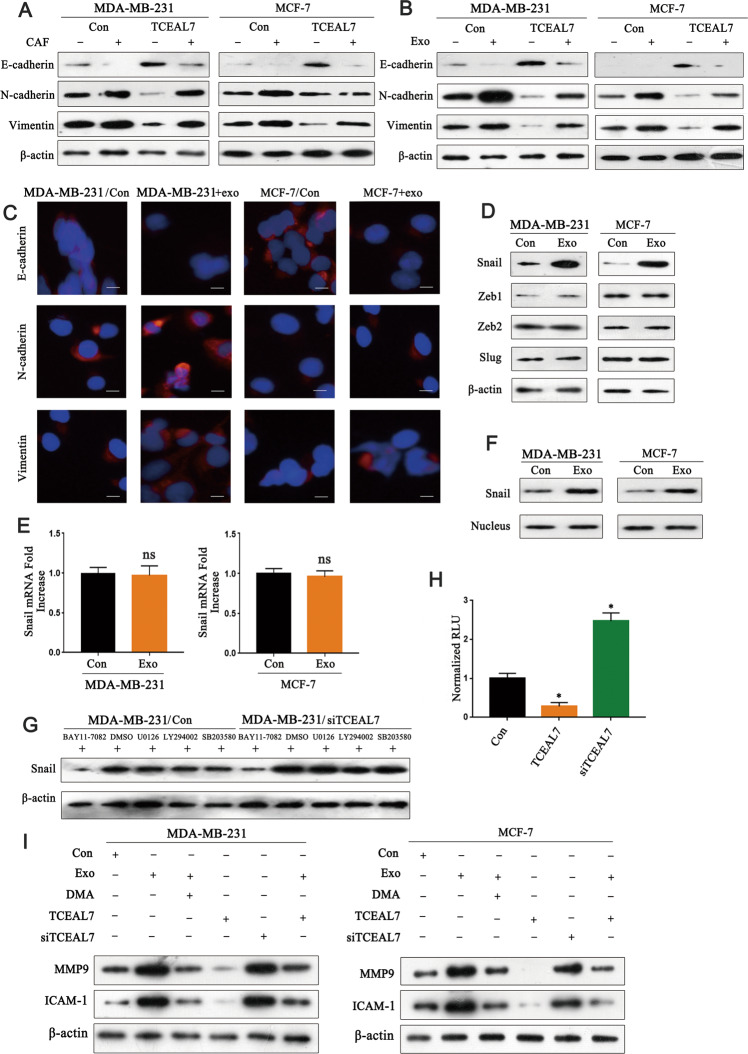
TCEAL7 reverse EMT by activating the NF-κB pathway. **A** Western blot analysis of the expression of E-cadherin, N-cadherin, and Vimentin in MDA-MB-231 cells after co-cultured with CAFs, and similar from MCF-7 cells (*n* = 3). **B** Western blot analysis the expression of E-cadherin, N-cadherin, and Vimentin in MDA-MB-231 cells after co-culturing with CAFs-derived exosomes, similar from MCF-7 cells (*n* = 3). **C** Cell immunofluorescence staining confirms Western blot results (*n* = 3). **D** The expression levels of upstream transcription factors of EMT such as Slug, Snail, Zeb1 and Zeb2 were detected by Western blot (*n* = 3). **E** The expression of Snail at the mRNA level in MDA-MB-231 cells and MCF-7 cells co-cultured with CAFs-derived exosomes (*n* = 3). **F** The expression of Snail at the protein level in MDA-MB-231 cells and MCF-7 cells co-cultured with CAFs-derived exosomes (*n* = 3). **G** Western blot to verify the effects of different inhibitors on Snail stability (*n* = 3). **H** Effection of TCEAL7 on NF-κB activation by luciferase assay (*n* = 3). **I** Western blot was used to detection of NF-kB target genes: matrix metalloproteinase 9 (MMP9), intercellular adhesion molecule-1 (ICAM-1) proteins (*n* = 3). * indicates *p* < 0.05. The measurement data were expressed using mean ± SD, and the experiment was repeated three times. Comparisons between two groups are analyzed by t-test; one-way ANOVA was used for multiple groups of data analysis.

EMT play key roles in mediating tumor cell invasion [27]. To determine whether CAFs is related to EMT of breast cancer cells, western blot results showed that MDA-MB-231 cells co-cultured with CAFs had the lower expression of E-cadherin and higher expression of N-cadherin and Vimentin than that of MDA-MB-231 cells co-cultured with NFs and we got similar results from MCF-7 cells (Fig. 6A, right). We further verified that tumor cells co-cultured with CAFs-derived exosomes expressed lower E-cadherin and higher N-cadherin and Vimentin than that of cells co-cultured with NFs-derived exosomes (Fig. 6B). In addition, the results of immunofluorescence staining were consistent with those of western blot (Fig. 6C). As mentioned before, our results showed that CAF-derived exosomes decreased the expression of TCEAL7 in BC cells. To explore the effect of TCEAL7 on EMT, we upregulated the expression of TCEAL7 in BC cells and found that the expression of E-cadherin was increased, whereas the expression of N-cadherin and Vimentin was decreased (Fig. 6A, left). Expression of E-cadherin, N-cadherin, and Vimentin was also validated using real-time PCR to show regulation at the transcriptional level, MDA-MB-231 cells co-cultured with CAFs or CAFs-exo had the lower mRNA expression of E-cadherin and higher mRNA expression of N-cadherin and Vimentin. However, the overexpression of TCEAL7 increase the expression of E-cadherin, whereas the expression of N-cadherin and Vimentin was decreased, and similar results from MCF-7 cells (Supplementary Figs. S6, 7). To further explore the upstream molecules that affected EMT, we measured the expression levels of several transcription factors in MDA-MB-231 cells. It was found that the Snail protein but not the Snail mRNA was significantly upregulated after co-cultured with CAFs-derived exosomes and the expression levels of other transcription factors (Zeb 1, Zeb 2, and Slug) were stable (Fig. 6D, E). Then we try to determine whether CAFs-derived exosomes were related to the Snail nuclear translocation event. As shown in Fig. 6F, the expression of Snail protein in the nucleus of MDA-MB-231 cells co-cultured with CAFs-derived exosomes was significantly higher than that of control MDA-MB-231 cells. Recent evidence revealed that TCEAL7 negatively regulates NF-κB pathway [28], as NF-κB could induce Snail promoter activity [29–31], then MDA-MB-231 and MDA-MB-231/siTCEAL7 cells were treated with specific inhibitors to determine which signaling pathway is involved in Snail stabilization. The results suggested that only NF-κB inhibitor (BAY11-7082) could significantly inhibit the stability of Snail (Fig. 6G). To analyze the effect of TCEAL7 on NF-κB activation, we transfected MDA-MB-231, MDA-MB-231/TCEAL7, and MDA-MB-231/siTCEAL7 cells with NF-κB luciferase reporter gene plasmid and found that the luciferase activity of MDA-MB-231/TCEAL7 cells was significantly lower than that of MDA-MB-231 cells while that of MDA-MB-231/siTCEAL7 cells was apparently higher than that of MDA-MB-231 cells (*P* < 0.05) (Fig. 6H). Studies have found that activating the NF-κB pathway by loss of TCEAL7 may be one of the mechanisms by which normal cells obtain proliferation and survival advantages. In order to study the mechanism of CAFs-exo regulation of TCEAL7 activity on invasion, and metastasis, we evaluated the expression levels of MMP9, and ICAM-1. The results showed that compared with the control group, CAFs-exo upregulated the expression of MMP9 and ICAM-1, and this effect was mimicked by the loss of TCEAL7. Overexpression of TCEAL7 reduces the expression of MMP9 and ICAM-1. In addition, overexpression of TCEAL7 can rescue the effects of CAFs-exo on MMP9 and ICAM-1 (Fig. 6I). Expression of MMP9 and ICAM-1 were also validated using real-time PCR to show regulation at the transcriptional level (Supplementary Fig. S8). Taken together, these results indicate that the TCEAL7 involved in invasion and metastasis progression by regulating the transcriptional modulation of NF-κB target genes., moreover, CAFs-exo can inhibit the role of TCEAL7 in this process. Together, these in vitro data indicate that TCEAL7 could reverse EMT by suppressing NF-κB pathway to suppress breast cancer cell invasion and metastasis.

### Exosome miR-18b induces tumor invasion and metastasis in mice

**Fig. 7:**
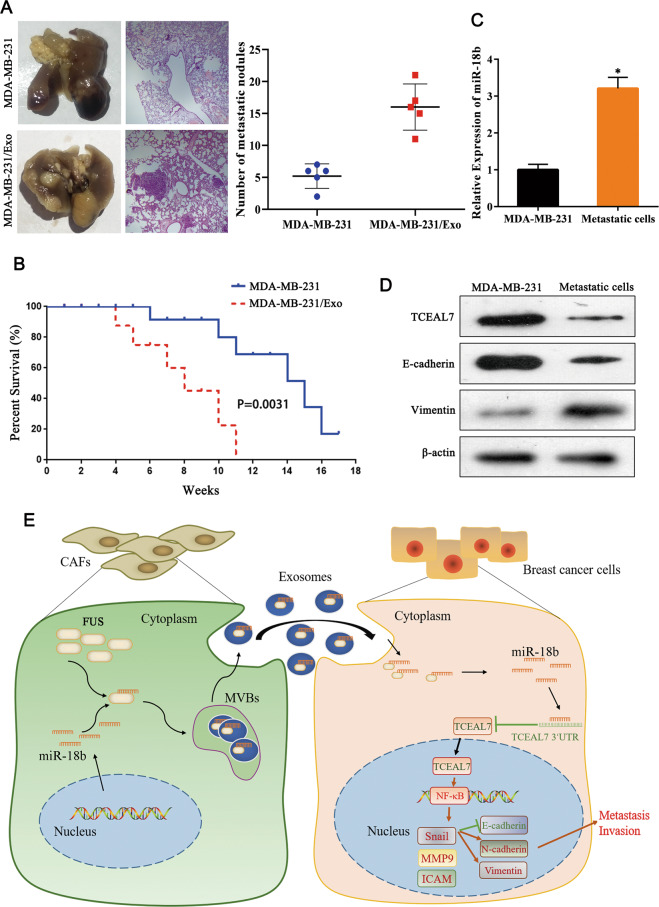
Exosome miR-18b induces tumor growth and metastasis in mice. **A** Comparison of pulmonary metastases in mice (*n* = 3). **B** Survival analysis showed that exosome miR-18b reduced survival rate in mice. **C** qRT-PCR detection of miR-18b expression in metastatic cells (*n* = 3). **D** Western blot indicated the expression of TCEAL7, E-cadherin, and Vimentin in mouse lung metastatic cells (*n* = 3). **E** A proposed model illustrating the role of CAF-derived exosomal miR-18b promotes BC cells invasion and metastasis by downregulation of TCEAL7. * indicates *p* < 0.05. The measurement data were expressed using mean ± SD, and the experiment was repeated three times. Comparisons between two groups are analyzed by t-test.

Finally, we did experiments in mice to further evaluate the roles of exosome miR-18b in breast cancer metastasis and survival situation. MDA-MB-231 cells and exosomal miR-18b were injected into mice simultaneously or separately through the tail vein. After six weeks, we found that mice of exosomes miR-18b group had more metastases in lungs than control group. Colonization in lungs was examined through H&E staining. As showed in Fig. 7A, the results showed that the number of metastatic tumor nodules in the lungs of exosomes miR-18b group was more than the control group. Meanwhile, the survival rate of exosomes miR-18b group was significantly lower than the control group as shown in Fig. 7B (*P* < 0.05). Then we separated cancer cells from the metastases of mice lung tissues and detected miR-18b, TCEAL7, E-cadherin, and Vimentin expression in lung metastatic cells. qRT-PCR results showed that in exosomes miR-18b group the content of miR-18b was higher than the control group, however, the expression of TCEAL7, E-cadherin, and Vimentin were significantly lower than the control group at the protein level (*P* < 0.05) (Fig. 7C, D). Additionally, Western blot analysis results showed (Supplementary Fig. S9) that the metastatic cells group had significantly increased expression of various genes that promote invasion (matrix metallopeptidase 2, matrix metallopeptidase 9) compared with the MDA-MB-231 group, whereas proliferation (Ki-67, PCNA) had no significant changes (*P* < 0.05). Taken together, these findings suggest that miR-18b-loaded exosomes had a cancer-promoting effect in the xenograft model. Finally, we provide a schematic diagram to reveal the biological role of the transcellular signaling pathway, comprising exo-miR-18b, FUS, TCEAL7, to promote BC cells invasion and metastasis (Fig. 7E).

## Discussion

Breast cancer is one of the malignant diseases that seriously threaten the life and health of women all over the world [32]. The tumor microenvironment is usually composed of tumor cells, extracellular matrix (ECM), stromal cells, immune cells, and cells from the blood and lymphatic system [33]. The composition of TME and the intercellular communication affect tumor progression, prognosis, and therapeutic effect [34]. Meanwhile, tumor cells can reprogram TME stromal cells to form the optimal tumor phenotype [35, 36].

Studies have shown that NFs can inhibit tumor progression [37]. Normal fibroblasts can be transformed into CAFs after co-culturing with cancer cells, promoting tumor progression through specific communication with cancer cells [38, 39]. CAFs can increase the proliferation of cancer cells by secreting stromal cell-derived factor 1 (SDF1) or produce ECM-degrading protease to affect cell invasion and movement [3, 40, 41]. This study compared the effects of CAFs and NFs on tumor cells and showed that CAFs promoted cell proliferation, migration, and metastasis.

Exosomes from different cell sources are extracellular vesicles enclosed by lipid bilayers that carry molecules such as miRNAs and proteins, which play an essential role in the communication between the tumor microenvironment and cancer cells. Chen et al. found that miR-500a-5p was upregulated in CAF and CAFs-derived exosomes and transferred from CAFs to cancer cells. miR-500a-5p promotes proliferation and metastasis of breast cancer cells by binding to ubiquitin-specific peptidase 28 (USP28) [42]. Exosomal miR-92 derived from cancer-associated fibroblasts promotes migration and proliferation of breast cancer cells, miR-92 can promote PD-L1 expression by enhancing occupation between YAP1 and PD-L1 enhancer regions to suppress immune cell function in breast cancer [43]. Breast cancer-derived exosomal survivin converts fibroblasts into myofibroblasts by upregulating SOD1. SOD1-overexpressing fibroblasts promote the proliferation and metastasis of breast cancer [44]. It has been reported that FAK signaling in CAFs regulates the CAFs-derived exosomes to promote breast cancer cell migration and metastasis [45]. In this study, we isolated exosomes from the culture medium of NFs and CAFs to explore their role and potential mechanisms in the development of breast cancer. By co-culturing of exosomes with breast cancer cells, we found that CAFs-derived exosomes had stronger effects on cancer cell proliferation, migration, and invasion.

MiRNAs can perform biological functions by binding to 3′UTR of target genes. There have been found that miR-18b is one of the differentially expressed miRNAs in multiple tumor types [26, 46, 47]. Miguel A et al. demonstrated that miRNA-18b is upregulated in breast cancer and promotes the migration of breast cancer cells [16]. However, the expression pattern and biological function, and underlying mechanism of miR-18b in breast cancer progression have not been fully elucidated. The present study indicated that miR-18b is significantly upregulated in CAFs-derived exosomes and the overexpression of miR-18b promotes cell invasion and migration. Moreover, exosomes miR-18b significantly promote tumor metastasis in vivo. Importantly, we further investigated how miR-18b was packed into exosomes. RBPs such as hnRNPA1, Ago2, and Y-box protein 1 are involved in exosomal miRNA transport by binding specific motifs [48–50]. Our data demonstrated that FUS plays a vital role in packaging miR-18b into exosomes by binding a specific motif (GGUG) of miR-18b. However, how MiR-18b is packaged into exosomes has not yet been fully elucidated. Therefore, further research is needed in the future to clarify these issues better.

TCEAL7 is a member of the transcription elongation factor A (SII)-like gene family and is relatively poorly reported. TCEAL7 was cloned as a pro-apoptotic nucleoprotein for the first time, and has been identified as an anti-oncogene and a cell death regulator. In addition, previous studies found that TCEAL7 was downregulated in various human tumors including ovarian cancer [18], but its specific roles in tumors have been rarely reported [21]. The initial finding of our bioinformatics analysis was that TCEAL7 is poorly expressed in breast cancer. In addition, our study unraveled that TCEAL7 was the target gene of miR-18b, the expression of TCEAL7 in breast cancer tissues was lower than that in normal tissues and negatively correlated with pathological grade.

NF-κB signaling pathway is widely activated in diverse human cancers [51]. Snail is recognized as a potent E-cadherin repressor and is a downstream target for NF-κB. The expression of Snail can be regulated by NF-κB through both transcriptional and post-translational mechanisms [52]. Studies have shown that miR-210-3p maintains the continuous activation of NF-κB signaling by targeting TNIP1 and SOCS1 and leads to EMT and invasion of prostate cancer cells [53]. Here, our data verified that TCEAL7 was negatively correlated with the expression of miR-18b in breast cancer tissues and was identified as the direct target gene of miR-18b in breast tumor cell lines. It is widely accepted that NF-κB induces a significant increase in snail expression, which leads to a remarkable decrease of E-cadherin-mediated intracellular adhesion, EMT, and metastasis/invasion of cancer cells. Consistently, our results revealed the role of NF-κB in the downstream pathway of miR-18b-TCEAL7 axis in breast cancer by demonstrating that TCEAL7 can inhibit NF-κB activation, thereby stabilizing Snail and ultimately inhibiting EMT. In addition, intercellular adhesion molecule 1 (ICAM-1) is a cell surface glycoprotein and adhesion receptor related to metastasis [54, 55]. Matrix metalloproteinase 9 (MMP9) is associated with the progression and invasion of malignant tumors [56, 57]. Our findings illustrate that CAFs-exo upregulated the expression of MMP9 and ICAM-1, overexpression of TCEAL7 can rescue the effects of CAFs-exo on MMP9 and ICAM-1, indicating CAF exosomes have a promoting effect on breast cancer cell invasion and metastasis, while TCEAL7 has an inhibitory effect on breast cancer cell invasion and metastasis.

In summary, our research indicates that the overexpressed miR-18b in CAFs-derived exosomes may promote the development and metastasis of breast cancer cells. Further study suggests that CAFs-derived metastatic exosomes miR-18b could activate NF-κB pathway by downregulating TCEAL7 expression in breast cancer cells to promote the malignant progression of tumors (Fig. 7). This study suggests that targeting miR-18b can be used as a new strategy to inhibit the development of breast cancer, and the research of CAF-derived exosomes carrying miR-18b has a positive effect on the therapy of breast cancer. Nevertheless, strict inclusion criteria should be developed and a larger cohort should be used in future studies to prove the definite diagnostic efficiency of miR-18b.

## Supplementary information

Supplementary information

Reproducibility checklist

## Data Availability

The datasets used and/or analyzed of this study are available from the corresponding author upon reasonable request.
