# Supplementary material for: Cancer-associated fibroblast-derived exosomal miR-18b promotes breast cancer invasion and metastasis by regulating TCEAL7
Source: Cell Death Dis. 2021 Dec 1;12(12):1120. doi: 10.1038/s41419-021-04409-w (PMC8636636; doi:10.1038/s41419-021-04409-w)
Supplement: Supplementary file 1 — Supplementary information [file 41419_2021_4409_MOESM1_ESM.pdf]

Cancer-associated Fibroblast-derived Exosomal miR-18b Promotes Breast Cancer Invasion and Metastasis by Regulating TCEAL7

Ziqian Yan, Zhimei Sheng, Yuanhang Zheng, Ruijun Feng, Qinpei Xiao, Lihong Shi, Hongli Li, Chonggao Yin, Hao Luo, Chong Hao, Wenhao Wang, Baogang Zhang

Supplementary information

Figure Legends

Supplemental Fig. 1. CAFs promotes the migration and invasion of MCF-7 cells. **(A)** The effect of CAFs and NFs on the migration of MCF-7 cells was analyzed by scratch experiment. **(B)** Invasion experiment showed the effection of CAFs and NFs on the invasion ability of MCF-7 cells (n=3). ** indicates $p< 0.01$; * indicates $p<0.05$.

Supplemental Fig. 2. CAFs derived exosomes enhance invasion and migration of MCF-7 cells. **(A)** Effects of exosomes on the invasion and migration ability of MCF-7 cells determined using Transwell assay (n=3). **(B)** Effects of exosomes from different fibroblast sources on the invasion and migration ability of MCF-7 cells analyzed by Transwell assays (n=3). ** indicates $p< 0.01$; * indicates $p<0.05$.

Supplemental Fig. 3. miR-18b is significantly upregulated in the exosomes of CAFs derived from breast cancer patients. **(A)** Heatmap of differential miRNA expression profiles of NFs and CAFs. The expression level of miRNA is hierarchically clustered on the y-axis, and CAFs or NFs are hierarchically clustered on the x-axis. Red means up-regulation; green means downregulation. **(B)** Analysis of expression levels of 10 most upregulated miRNAs in exosomes derived of NFs and CAFs using real-time PCR

(n=3).

Supplemental Fig. 4. The up-regulated expression of miR-18b in exosomes enhanced migration and invasion of MCF-7 cells. (A) qRT-PCR detected miR-18b expression in MCF-7 cells co-cultured with NFs and CAFs-derived exosomes (n=3). **(B)** Transwell assays detected the effect of miR-18b on the ability of MCF-7 cells in migration and invasion (n=3). ** indicates $p< 0.01$; * indicates $p<0.05$.

Supplemental Fig. 5. miR-18b has no effect on cell proliferation. Comparison of cell proliferation in miR-18b mimics/MDA-MB-231 and Con/MDA-MB-231.

Supplemental Fig. 6. Overexpression of TCEAL7 can reverse EMT caused by CAFs. (A-C) Expression of E-cadherin, N-cadherin and Vimentin in MDA-MB-231 cells after co-cultured with CAFs was validated using real-time PCR to show regulation at the transcriptional level, and similar from MCF-7 cells **(D-F)** (n=3). ** indicates $p< 0.01$; * indicates $p<0.05$

Supplemental Fig. 7. Overexpression of TCEAL7 can reverse EMT caused by CAFs-derived exosomes. (A-C) Expression of E-cadherin, N-cadherin and Vimentin in MDA-MB-231 cells after co-cultured with CAFs-derived exosomes was validated using real-time PCR to show regulation at the transcriptional level, and similar from MCF-7 cells**(D-F)** (n=3). ** indicates $p< 0.01$; * indicates $p<0.05$

Supplemental Fig. 8. **CAFs-derived exosomes increased the expression of MMP9 and ICAM-1 at the mRNA level.** **(A)** Expression of MMP9 and ICAM-1 in MDA-MB-231 cells was validated using real-time PCR at the mRNA level, and similar from MCF-7 cells **(B)** (n=3). ** indicates $p< 0.01$; * indicates $p<0.05$

Supplemental Fig. 9. **CAFs-derived exosomes increased the expression of Ki-67, PCNA, MMP2 and MMP9.** Expression of Ki-67, PCNA, MMP2 and MMP9 was validated using Western Blot at the protein level(n=3).

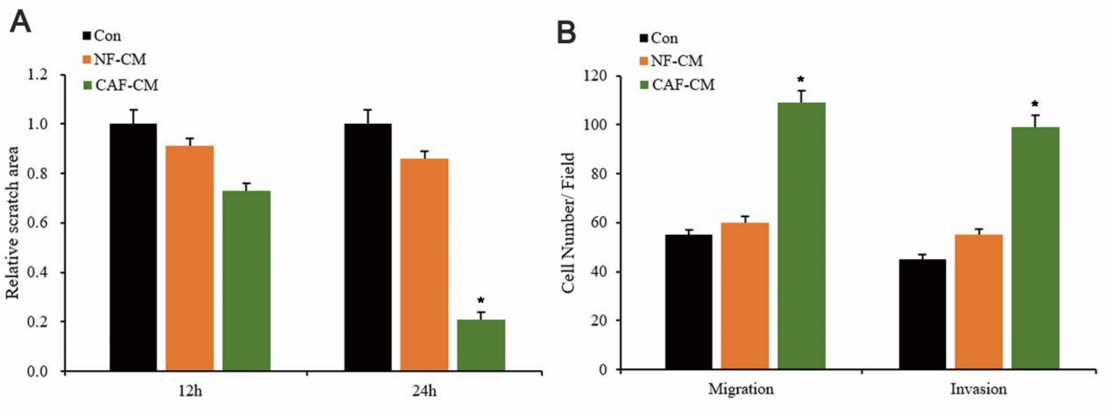

Supplemental Fig. 1

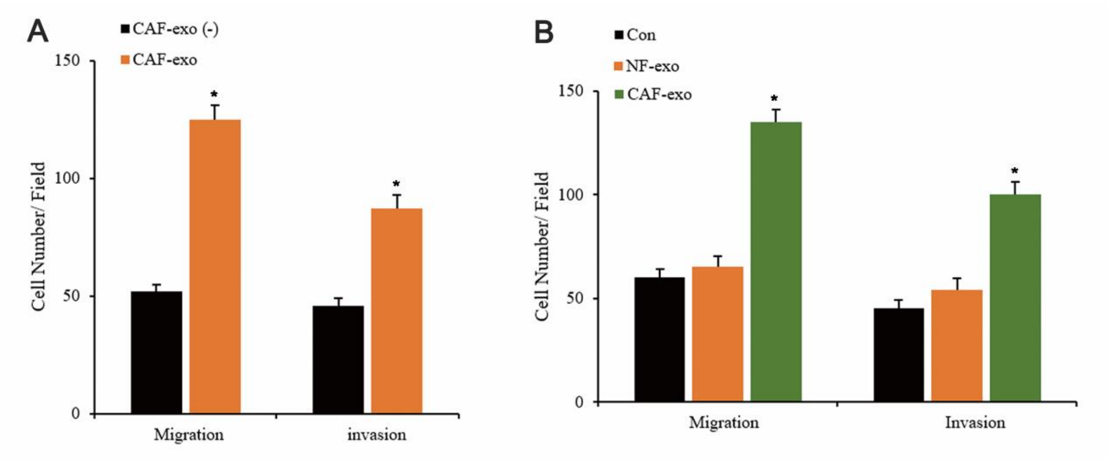

Supplemental Fig. 2

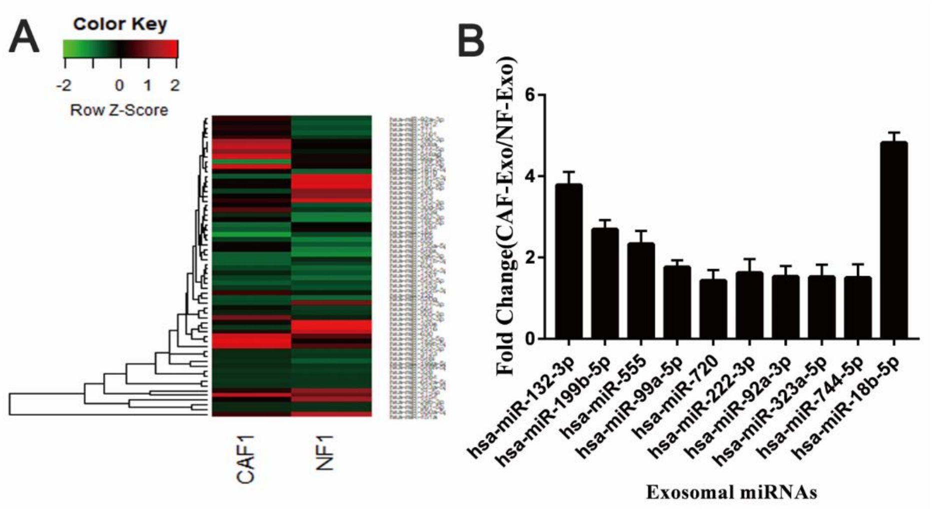

Supplemental Fig. 3

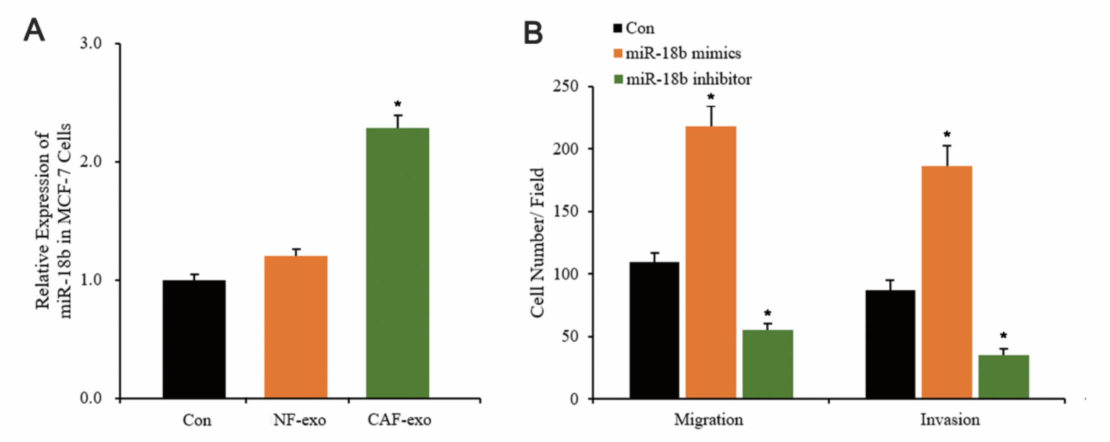

Supplemental Fig. 4

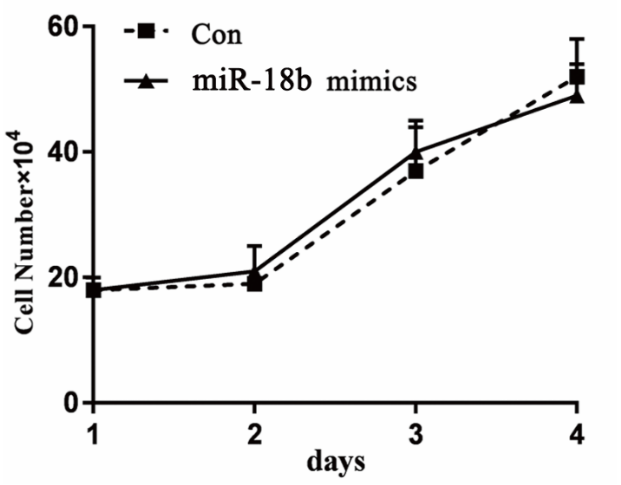

Supplemental Fig. 5

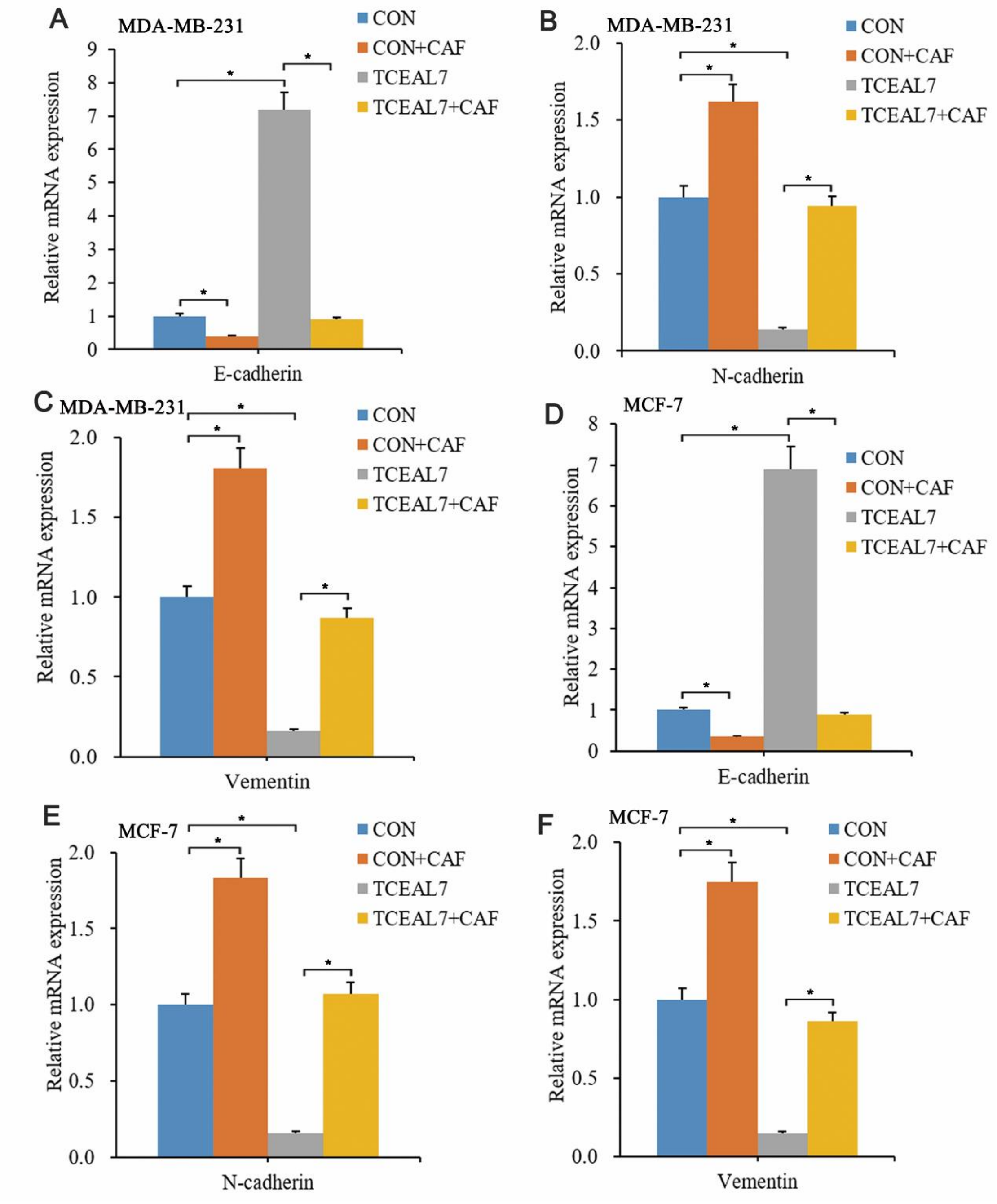

Supplemental Fig. 6

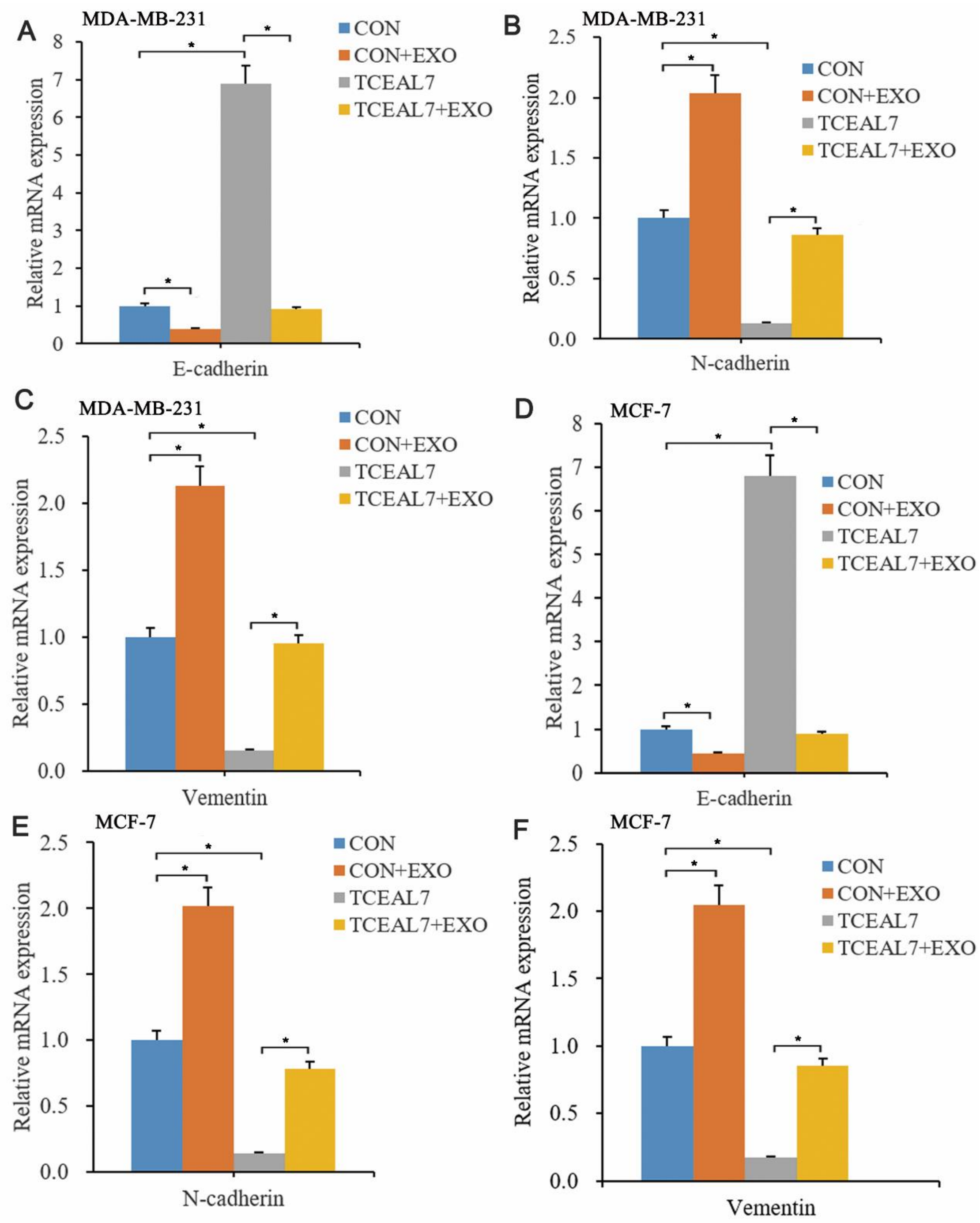

Supplemental Fig. 7

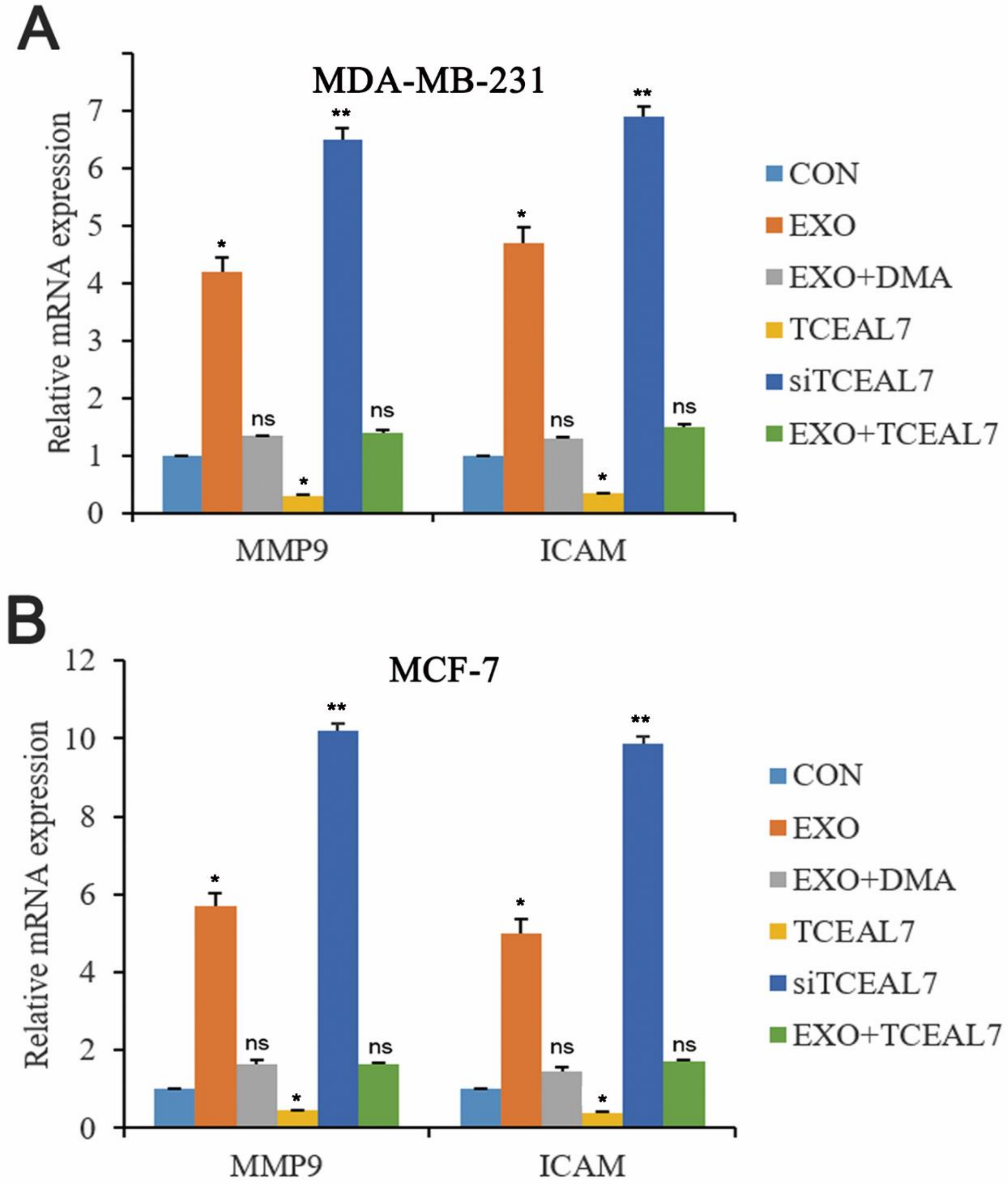

Supplemental Fig. 8

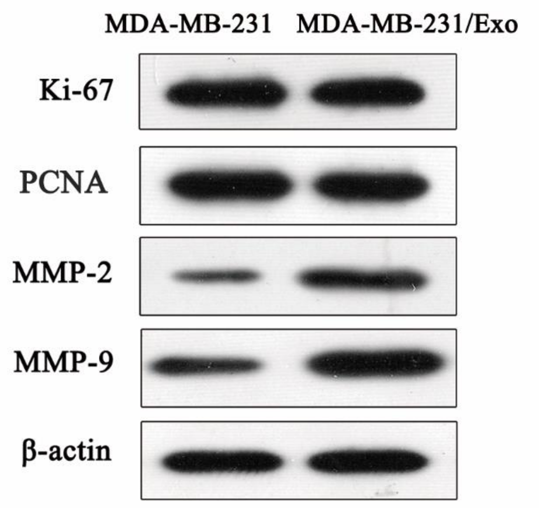

Supplemental Fig. 9
